# “It’s what midwifery is all about”: Western Australian midwives’ experiences of being ‘with woman’ during labour and birth in the known midwife model

**DOI:** 10.1186/s12884-018-2144-z

**Published:** 2019-01-14

**Authors:** Zoe Bradfield, Yvonne Hauck, Michelle Kelly, Ravani Duggan

**Affiliations:** 1 School of Nursing, Midwifery and Paramedicine, Curtin University, GPO Box U1987, Bentley, WA 6845 Australia; 2 School of Nursing, Midwifery and Paramedicine, Curtin University King Edward Memorial Hospital, Subiaco, Australia

**Keywords:** With woman, Midwives, Continuity of care, Known midwife model, Qualitative research, Phenomenology

## Abstract

**Background:**

The phenomenon of being ‘with woman’ is fundamental to midwifery as it underpins its philosophy, relationships and practices. There is an identified gap in knowledge around the ‘with woman’ phenomenon from the perspective of midwives providing care in a variety of contexts. As such, the aim of this study was to explore the experiences of being ‘with woman’ during labour and birth from the perspective of midwives’ working in a model where care is provided by a known midwife.

**Methods:**

A descriptive phenomenological design was employed with ten midwives working in a ‘known midwife’ model who described their experiences of being ‘with woman’ during labour and birth. The method was informed by Husserlian philosophy which seeks to explore the same phenomenon through rich descriptions by individuals revealing commonalities of the experience.

**Results:**

Five themes emerged 1) Building relationships; 2) Woman centred care; 3) Impact on the midwife; 4) Impact on the woman; and 5) Challenges in the Known Midwife model. Midwives emphasised the importance of trusting relationships while being ‘with woman’, confirming that this relationship extends beyond the woman – midwife relationship to include the woman’s support people and family. Being ‘with woman’ during labour and birth in the context of the relationship facilitates woman-centred care. Being ‘with woman’ influences midwives, and, it is noted, the women that midwives are working with. Finally, challenges that impact being ‘with woman’ in the known midwife model are shared by midwives.

**Conclusions:**

Findings offer valuable insight into midwives’ experiences of being ‘with woman’ in the context of models that provide care by a known midwife. In this model, the trusting relationship is the conduit for being ‘with woman’ which influences the midwife, the profession of midwifery, as well as women and their families. Descriptions of challenges to being ‘with woman’ provide opportunities for professional development and service review. Rich descriptions from the unique voice of midwives, provided insight into the applied practices of being ‘with woman’ in a known midwife model which adds important knowledge concerning a phenomenon so deeply embedded in the philosophy and practices of the profession of midwifery.

## Background

Midwives being ‘with woman’ is the central construct of the Professional Philosophy Statement implemented by the Australian College of Midwives (ACM). The Statement confirms how this phenomenon guides the philosophy, relationships and practices of midwives [1]. Being ‘with woman’ is embedded in professional codes and standards guiding Australian midwifery practice [2–4]. The importance of being ‘with woman’ is also referenced in pivotal professional publications from peak midwifery bodies internationally including, Royal College of Midwives in the United Kingdom (UK) [5] and the New Zealand College of Midwives [6].

The significance of the midwife-woman relationship is consistently referred to in literature addressing midwives’ being ‘with woman’ [7–11] and; working in partnership with women is central to the professional philosophy statement authored by the International Confederation of Midwives [12]. However, evidence referring to midwives being ‘with woman’ is primarily sourced from theoretical writings from midwifery leaders [13–15] and studies focusing upon women’s experiences of being cared for by midwives [16–18]. Writings from midwifery leaders worldwide emphasize the importance of being ‘with woman’ and articulate the practice attributes that stem from this philosophical basis such as woman –centred care and working in partnership with women [6, 13, 19, 20]. Studies exploring women’s experiences have shown that midwives being ‘with woman’ provides a platform for empowerment and enhances confidence in childbearing women [10, 21]. Findings of further research confirms that women’s experiences and outcomes during labour and birth are improved when continuity of care is provided by a known midwife and is facilitated by the midwife-woman relationship developed in this model [17, 22, 23].

Despite the centrality of being ‘with woman’ to the profession of midwifery, a recent integrative review found that there has been no research that has specifically explored the phenomenon from the perspective of midwives [11]. This research forms one component of a series of studies undertaken in other models where care is provided by unknown midwives (standard public care) [24] or unknown midwives and known obstetricians (private obstetric models) [25] which revealed the importance of building a connection with the woman and the centrality of ‘relationship’ to the practices of being ‘with woman’. In light of the contribution of the midwife-woman relationship to being ‘with woman’ [13, 20, 26], the intersection between the models that enable care during labour and birth by a ‘known midwife’ and the ‘with woman’ phenomenon warrants exploration.

The aim of this study was to explore Western Australian midwives’ experiences of being ‘with woman’ during labour and birth in the context of a model that facilitates care by a known midwife.

## Methods

### Research setting

In Western Australia (WA), the opportunity for women to receive care in labour and birth by a known midwife is available through a number of existing midwifery continuity of care programs. The Community Midwifery Program (CMP) is a publically funded midwifery-led service that provides continuity of care from a known midwife from 16 weeks of pregnancy, throughout labour, birth and for up to 2 weeks post birth. Women who live within a 50 km radius of the Perth Central Business District, and meet the criteria may self-refer and elect to birth in their own home, at WA’s only Birth Centre located adjacent to WA’s only tertiary maternity service or, at a participating public hospital [27, 28].

Midwifery Group Practices (MGPs) provide care to pregnant women in a group of (4–6) midwives. The woman has the opportunity to meet with and receive antenatal care by each of the midwives in the group, with a commitment that two of the midwives will be present for the birth. MGPs offer care to women booked to birth in participating public hospitals, the woman’s home [28] (1 rural site) as well as the state’s only birth centre. Within WA, MGPs are coordinated differently at each site [29]. Women are cared for primarily by midwives and where required, in collaboration with medical specialists according to the woman’s health needs, policy of the health agency and in conjunction with the National Midwifery Guidelines for Consultation and Referral [30].

A final option is to employ the services of a midwife in private practice where women recruit and contract with individual midwives to provide care across their childbearing experience. Women using this model usually birth in their own homes although there are a number of midwives in private practice who have access agreements with public hospitals that may enable admission under the care of the midwife as explained in the information published by the Department of Health in Western Australia [28]. Each of these three models of care (CMP, MGP and Midwives in private practice) enable the woman to receive care during her labour and birth by a known midwife.

Phenomenological research explores the lived experience of participants in a way that relays the essences and meanings of a phenomenon [31]. This study employed a framework for descriptive phenomenological research developed by Giorgi [32, 33]. Giorgi’s work is informed by the Husserlian philosophy of Phenomenology which focusses on descriptions of the same phenomenon as it manifests itself to different individuals and reveals commonalities of the experience [32, 34]. A descriptive phenomenological methodology offers an ideal opportunity to gain further insight into the applied practices or experiences of the ‘with woman’ phenomenon from the perspective of midwives working in a ‘known midwife’ model as it uncovers the constituents of phenomena that have not been conceptualised by prior research [35–37]. Descriptive phenomenology is also useful to gain an understanding of the eidetic structures of a phenomenon in a way that “… neither adds nor subtracts from the invariant intentional object arrived at, but describes it precisely as it presents itself” [38].

Purposive sampling of midwives working in one of the ‘known midwife’ models described above led to snowballing and the recruitment of midwives into this study. Snowball sampling is useful when information gathered from ‘purposefully sampled’ participants connects the researcher to other potential participants [31]. The inclusion criteria were: midwives who had provided labour and birth care in a model that facilitates care by a known midwife in the last 12 months to mitigate recall bias. Ten female midwives participated in the study ranging in age from 35 to 57 years with between 4 to 34 years of midwifery experience. All had previously worked in public hospital based maternity systems that offered fragmented care where care was provided to labouring women not known to them. Demographic profile is presented in Table 1.

**Table 1:** Demographic profile (*n* = 10)

Demographic variables	Number of participants
Age	
30 to 40	2
41 to 50	5
50 to 60	3
Years of experience as a midwife	
< 5 years	1
5 to 10 years	3
11 to 15	3
16 to 20	
21 to 25	1
26 to 30	1
31 to 35	1
Method of midwifery education	
Hospital – based diploma	3
Undergraduate midwifery degree	3
Postgraduate midwifery qualification	4
Country of midwifery education	
Australia	6
United Kingdom	3
New Zealand	1
Previous midwifery model	
Public hospital non-continuity model	10
Private hospital obstetric model	1
Current midwifery model	
Privately practicing midwife	2
Midwifery group practice	8
Total	10

### Data collection

In depth interviews were conducted, digitally recorded and transcribed verbatim. Midwives were asked to describe their experiences of being ‘with woman’ during labour and birth and to reflect on any intersection on the phenomenon in the context of the ‘known midwife’ model. In- depth interviews lasting on average, over an hour long were conducted between December 2016 and May 2017 by the first author (ZB) at a time and place convenient to the midwives. The interviewer, a midwife academic, was known to two midwives, however the potential for influence on participant responses was limited as the researcher was not working in a clinical setting with any of these midwives. Throughout the interview process, the practice of phenomenological reduction was adopted which involves suspending personal assumptions or prior knowledge of being ‘with woman’ and considering the phenomenon as it was described [32, 33]. Field notes and a research journal were maintained, which offered the opportunity to bracket any pre-existing thoughts and to reflect post-interview [39]. Pre-brief and de-brief with the rest of the research team (YH, MK, RD) was also used as a strategy to enhance bracketing. Midwives were offered the opportunity to send additional comments after the interview to the researcher. Two midwives provided further comments via email and two via a phone call. Data saturation was becoming apparent after eight interviews and a further two interviews were performed so the research team were able to confirm further data was not generating new information [40].

### Data analysis

Data analysis was guided by Giorgi’s four stage phenomenological approach: (1) data immersion, (2) dividing data into parts, (3) organisation and transformation of data and (4) expressing of the constituents of the phenomenon [32]. Transcripts were read and re-read with repeated listening of the audio recording to facilitate immersion in the data. Conceptualisations known as ‘meaning units’ were extracted. Qualitative data analysis software NVivo (version 11) was employed for classification and arrangement of data. Step three facilitated organisation and expression of data into the language of the profession. As participants described their experiences, the ways of experiencing the phenomenon were revealed. Here, statements were transformed from everyday language to concepts that Giorgi [32] maintains, are revealed through the researcher’s disciplinary intuition. The final step involved developing the key constituents (expressed as themes) and essences (expressed as sub- themes). These concepts were supported by direct quotations from interviews which are indicated in italics with a unique identification code (P1 to P10) to ensure confidentiality of the participants. To enhance clarity and for brevity, non-italicised words in square brackets [] have been inserted by the researcher to provide context to descriptions and; where words were omitted this is indicated by an ellipsis (…). The first author conducted the interviews and analysed all transcripts. Each transcript was analysed by at least two members of the research team. The team then met to discuss preliminary findings and consensus was reached around final themes and subthemes adding rigor to the analysis. Using NVivo 11, a Word Cloud was also generated from the interview transcripts which highlights the midwives’ language when sharing their experience of being ‘with woman’. Word Clouds are increasingly used in qualitative research as a way of enhancing transparency and drawing attention to the dominant narrative evidenced by more frequent words appearing in bold and larger font [41–44].

## Results

**Fig. 1:**
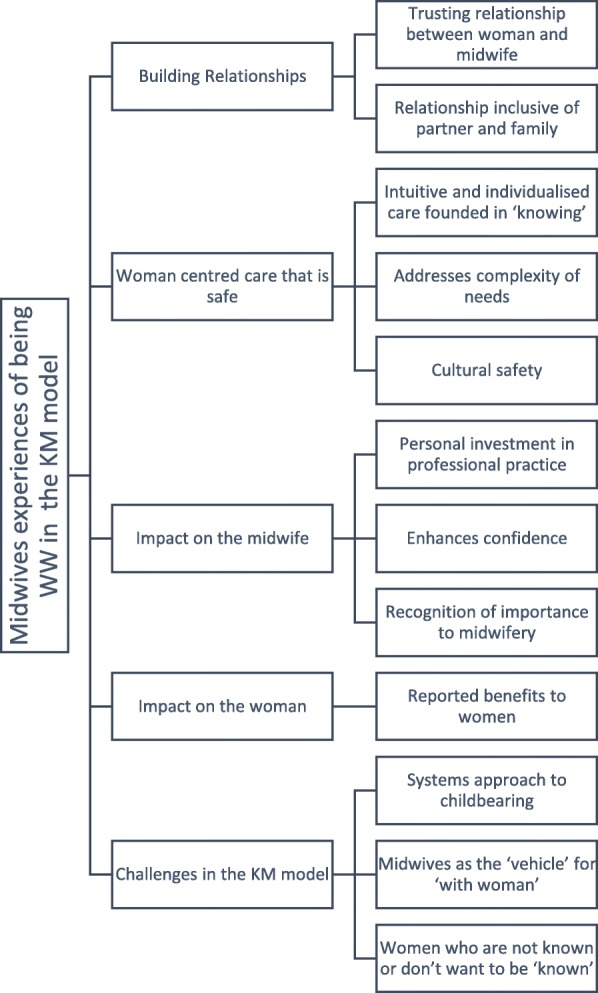
Themes and subthemes: Western Australian (WA) midwives’ experiences of being ‘with woman’ in known midwife (KM) models

**Fig. 2:**
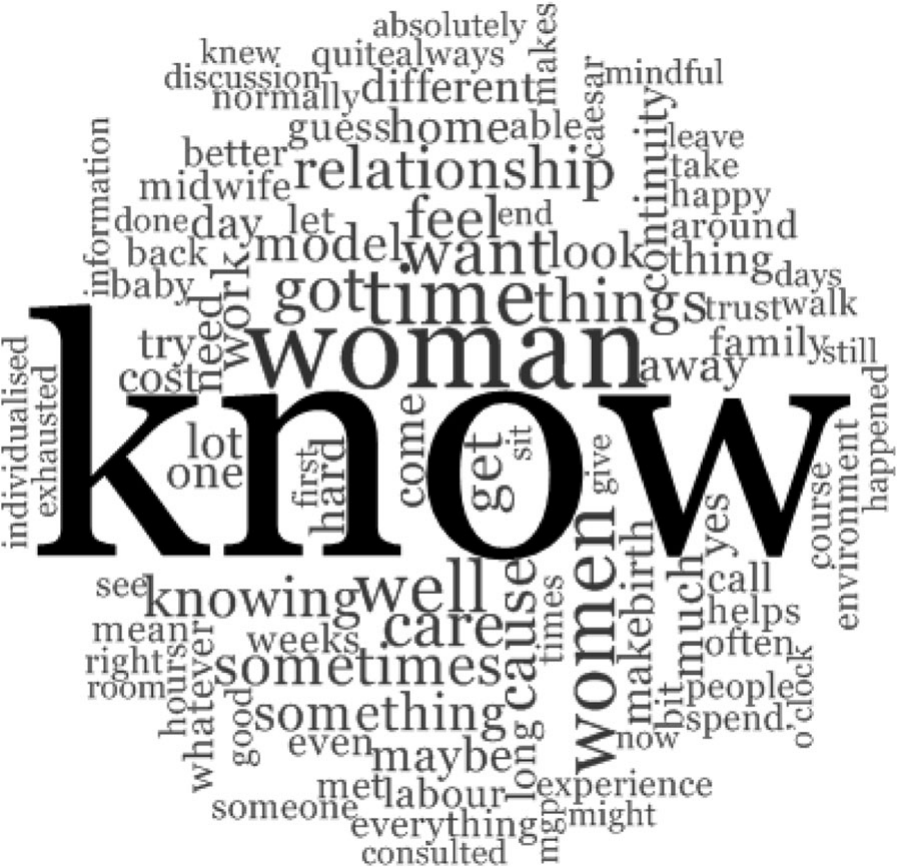
Word Cloud generated from all interview transcripts using NVivo® 11

Analysis of midwives’ experiences of being ‘with woman’ in the context of a ‘known midwife’ model revealed rich descriptions that offer insight into how the model intersects with the phenomenon. Five main themes were identified, 1) Building relationships; 2) Woman centred care that is safe; 3) Impact on the midwife; 4) Impact on the Woman; 5) Challenges in the ‘known midwife’ model along with corresponding subthemes (Fig. 1). The dominant narrative presented during the midwives’ interviews as illustrated in the Word cloud (Fig. 2) complements the identified themes.

### Building relationships

#### Trusting relationship between woman and midwife

Midwives working within the ‘known midwife’ model shared their experience of developing a professional relationship with women as a consequence of working closely together for months. Descriptions were shared around how working with women in the intrapartum period was based upon an established relationship that enabled the process of being ‘with woman’. Midwives were asked to reflect on the intersection of the model and being ‘with woman’ in the intrapartum period. However, when sharing their experiences, midwives consistently referenced care provided across the childbearing continuum suggesting that in this model, being ‘with woman’ cannot be isolated to intrapartum care and is integrated across the childbirth continuum.

Whilst providing care during labour and birth, this established relationship characterised by trust is a significant element of being ‘with woman’. As one midwife suggested

… *you have that trust, a real trust in each other that was developed over time that you both rely on actually. She trusts you to make the right call and you trust her for her body to work efficiently and do what is necessary in the space that you’ve created (*P9).

This trusting relationship, sometimes conveyed as a partnership or professional friendship, was acknowledged by midwives as the key to the success to being ‘with woman’ during labour and birth in the ‘known midwife’ model: *I think first and foremost it’s the relationship … it becomes close to a friendship type of relationship … a respectful partnership and relationship between the midwives and the women* (P2).

Being able to provide woman - centred care was central to the ‘knowing’ created as a result of relationship-building during pregnancy*.*

*It’s like you’ve got that friendship already before you go in the room … as soon as you walk in the room the atmosphere’s different. We know each other and it’s relaxed and it’s, happy … my experience on MGP has been much better of having that sense of being with woman because I’ve had that continuity* (P4).

Midwives felt the relationship that developed, enhanced their ability to connect with the woman and her journey rather than being associated with a set of clinical tasks: *Your relationship with the woman grows like the baby grows: It’s a journey you make as well with the woman it’s not just the woman and her family but it’s really the partnership with them* (P7). Building the relationship during pregnancy contributed to the commitment and ‘knowing’ required to offer support during labour and birth: … *having that journey beforehand that helps you feel that commitment and that onus to do well … in birth I guess for that woman* (P8).

#### Relationship inclusive of partner and family

When describing the importance of relationship building, the midwives clarified that although the primary relationship is with the woman, a relationship also developed with the partner and other family members: *It [relationship] improves it [being with woman] because of the knowledge, because of knowing the family, knowing where they come from, know where they live, know what their background is … so that helps you antenatally but also for labour* (P7). The relationships built across pregnancy connected the midwife to their journey and allowed the midwife to situate the woman and her family at the centre of the care experience.

### Woman centred care is safer

#### Intuitive and individualised care founded in ‘knowing’

Building trusting relationships facilitated a deep ‘knowing’ of the woman and her significant other, which enhanced the midwife’s ability to offer intuitive individualised care: … *you’ve had the opportunity to have a discussion as to exactly what they want from their experience before you’re meeting a woman in labour who possibly can’t communicate that as well* (P6). Multiple examples were offered to illustrate how the relationship and deep level of knowing what the woman wanted for her birthing experience facilitated meeting her needs as they evolved across the intrapartum period. Interactions described during the care experience revealed how intuitive care was possible.

… *it was just literally eye contact, me knowing that lady, knowing what she wanted, where she wanted to be … watching her body, watching what she was doing … I could pick up on that and jump in when it was relevant* (P4).

Another midwife characterised this process as being in tune with the woman, almost like a reciprocal dance where the midwife could respond appropriately to the woman’s needs.

*You’ve had plenty of time and you know what her worries are but you also know what her insecurities are, you also know what her medical problems are and how you can look for that … you’re in tune with all of that it just forges the path forward and just makes it kind of clear and open so she’s able to walk that path with you by her side having that awareness* (P9).

A final example reflects how the midwife can facilitate a woman’s individual path in choosing a different course of action than might be initially recommended: … [*if] that approach is not going to work for her … I’ll try one other thing … I think if you’re a midwife who is working* ***‘****with woman’ … you will become the midwife who writes ‘declined’* (P2).

Midwives shared how being ‘with woman’ helped them to provide care reinforced by a sense of safety. Stories revealed how the relationship founded in trust and knowing of the woman’s health and preferences plus her family situation enabled them to feel more confident in their care:

*Working with the woman in a continuity of care model I feel safer as a midwife. I feel the knowledge I have of that woman, of her partner, of her needs, because I know her so well I’m safer* (P1). Another midwife noted:

*I feel safer looking after women who I know because I know their history very well. I know them inside out. I know what their normal situation is, what their medical history is; little things to look out for and know when to move, when to kind of change direction, when to think ok now we need to do something different* (P9).

#### Addresses complexity of needs

Midwives described how developing a close relationship assisted them to feel better equipped to care for a labouring woman whose pregnancy was complex from the outset or changed during pregnancy. Knowing the woman, her clinical issues plus her hopes and plans for her birth experience enhanced midwives’ ability to be ‘with woman’ and support the woman’s choices. Having time within the ‘known midwife’ model to attend obstetric consultations and facilitate authentic collaboration with care decisions put the woman at the centre of the care experience.

… *particularly if you’ve got women that have complex issues, you’ve got a full 4, 5 months of being with them to learn more about that problem rather than trying to be thrown in the deep end with someone in labour … you know their story, you know what they want, you get to spend more time with them, physically supporting them as opposed to have to sit there and read through their volume of notes to find out why does this woman not want this* (P5).

#### Cultural safety

The midwife and woman relationship characterised by understanding, knowing and trust facilitated care that was responsive and respectful to women and families from diverse ethnic and cultural backgrounds. This relationship fostered culturally safe care. As one midwife recounted:

*I work a lot with Indigenous women, facilitating those relationships during pregnancy has made birthing so much more easier and … opens you up to the community because they know who you are and who you represent (P3).* This trusted, respectful relationship broadens the traditional view of cultural safety that focuses on individuals from ethnic minority groups. One midwife noted that being ‘with woman’ requires an approach that considers each woman’s journey: … *this is her journey … cultural safety is really about not just different ethnicities it’s also about … other people’s experience or other people’s lives (P7).*

### Impact on the midwife

Midwives’ experiences of being ‘with woman’ during labour and birth in the ‘known midwife’ model revealed the impact of the phenomenon on midwives and the midwifery profession.

#### Personal investment in professional practice

Midwives spoke of the emotional impact that being ‘with woman’ has on them which requires self-awareness as participant (P4) shared: *If you’ve got midwives who do acquire the skill of emotional intelligence … they definitely have the ability to be with woman more*. The process of being ‘with woman’ engaged not only midwives’ professional identity but also connected at a personal and individual level: *It’s the emotion that I invest in that woman … and it’s not just of her it’s of her and her partner and other children, it’s very, very, very personal* (P1).

Personal investment in a professional practice was an act of personal and professional integrity which enabled midwives to be true to themselves and not just to the women … *without knowing the women, it just wouldn’t be the same either so unfortunately it’s all or nothing from me … if I’m going to be around birth … I just can’t see it any other way (P8).*

Midwives confirmed their commitment to working in a model that respects both midwives and women. One midwife highlighted these outcomes as:

*I know the hours I spend looking after women in the continuity of care model, if I spent those hours in a medical model … I would earn a lot, financially I would be so much better off but I know I would hate myself* (P1).

Midwives acknowledged that being ‘with woman’ in the ‘known midwife’ model did come at a ‘cost’. *There’s a cost in terms of sleep and exhaustion and family time and being on call, yes there’s lots of costs to do that (P8).* The connectedness that develops through the relationship results in an investment that sees midwives riding the highs and lows with the women.

*Sometimes you take it home at the end of the day it can be draining … the hours are unsociable, they’re often long. You know it can be physically and emotionally draining (P5)* but then also note: … *it’s more than made up for when I work in a model that like I’m in now … when you’ve had that full experience from beginning to end with a family … It definitely makes up for it* (P5).

#### Enhances confidence

Midwives reported a sense of confidence and security that arose from being ‘with woman’ during labour and birth in the context of the trusting relationship that develops in the ‘known midwife’ model. The confidence and security develops from the mutually trusting relationship and facilitates an environment where midwives have freedom and space to observe and respond to the woman’s needs *Wow that worked, ok we’ll do that next time …* [*if] that kind of situation presents I can do that again … So because I get the opportunity to practice I get … to see it work* (P2). The relationship that ‘gives’ to the woman also ‘gives’ to the midwife:

… *being able to recognise a situation and change it enough to help her achieve what she wants to gives you almost as much confidence as having the baby yourself you know … So I think that [being with woman] would give midwives more confidence to speak out and I think it’s important for women (P2).*

#### Recognition of importance to midwifery

There was unequivocal agreement that being ‘with woman’ is an important part of midwifery practice: *Absolutely, 100% no doubt it’s [being with woman] what you do the work for, you know* (P6). There was caution expressed that the skill of being ‘with woman’ is at risk of being lost: … *it’s important for midwives because lots of midwifery skills are being degraded or down-graded or lost* (P2). The importance of being ‘with woman’ extended to not just the practice of midwifery but to the very identification of what a midwife is: … *it’s absolutely essential because without it [with woman] you’re not a midwife* (P9).

The reward and fulfilment of being ‘with woman’ during labour and birth was acknowledged as a sustaining force that kept midwives engaged and committed despite the costs and challenges: … *you’re in that space with this woman and it’s really personal and it’s really intimate and that’s really special … then it’s rewarding … like, that’s what it’s about* (P10). The relationship that develops in this model enhanced midwives’ ability to practice in accordance with their professional philosophy contributing to job satisfaction.

… *my experiences of being with woman are better for me on MGP … to know I’ve had that with woman … the fact that I’ve made that difference is really, gives me really good job satisfaction (P4).* This is echoed by another midwife … *it’s [being with woman] why you do the job … I feel I get more out of my job when I have known them before (P5).*

Midwives acknowledged the privilege of working with women and their families: *I was talking to my sister last night about how privileged our job is and that you share in the most amazing moments* (P6) and that this sustains a commitment to the profession: *I can’t really imagine doing anything else* (P3).

### Impact on the woman

Midwives offered insights into their observations of the benefits that women might experience based on the feedback from women they had cared for.

#### Reported benefits to women

Midwives reported that ‘with woman’ care during labour and birth improves women’s birth experiences by enhancing respect and dignity**.** *The feedback we get from the woman is that they felt that they were treated with respect, dignity … not just being that clinician, it’s about actually being decent to people* (P3). Midwives shared how women valued the guidance that was provided:

… *the other thing women say is, you knew exactly what to say at the right times* (P6). According to midwives, women also reflected upon the relationship and how it sustained them during times of difficulty *I get a lot of feedback from women that say ‘oh my God that’s what got me through* (P6). Being ‘with woman’ in the context of the professional relationship contributed to women sharing with their midwife: *She (the woman) said ‘I felt like I was in control, I felt like I made decisions’ … she said to me ‘I never thought I’d have a relationship with my midwife like I have with you’* (P6). Midwives observed women being empowered: *That’s what you’ve done by being with woman … she will labour efficiently and she’ll feel so proud of herself afterwards to have done it under her own steam* (P9).

Midwives also shared how women compared their experiences based upon exposure to models of care where the midwife is not known to them.

Women who have had experience in both models will actually tell you that there is a difference. It was so important for her to achieve a VBAC [Vaginal Birth after Caesarean]. It couldn’t have actually worked out any better … She says ‘I cannot even begin to tell you the experience’, she says ‘they were incomparable … the whole experience, but particularly the birth’ (P3).

For many women, the stories shared with midwives confirm how the relationship with the midwife provided a source of healing and reconciliation of previous traumatic birth experiences: *She was very vocal about that* [previous experience] *and she was like ‘that was just such a healing birth for me’ … That to me was what being with woman was about* (P5).

### Challenges in the known midwife model

#### ‘Systems’ approach to childbearing

The ‘systems’ approach to maternity services that favour standardisation over individualised care can present a barrier to being ‘with woman’ during labour and birth. Midwives reflected on their previous experiences of being ‘with woman’ during labour and birth in other models, and some, from other countries. As one midwife noted:

… the lack of understanding on the establishment side and the medical side of midwifery-only care ... hospitals’ lack of willingness to work with midwives that choose to work in this model is a really big inhibitor … it’s worked … in the UK, where the medical obstetric profession and midwifery are very much on an even level and they’ve worked together forever … we’re all her carer, so long as she gets the right care that’s what matters. I still find it absolutely frustrating the way the system dictates to women you will or won’t have and that midwives can or cannot … that’s the biggest inhibitor to working in this model (P1).

Midwives shared how the requirement for medical involvement in the care of women impacts the relationship between midwives and women … *within the hospital system, that relationship constantly gets eroded and women must be aware of that* (P2).

Midwives felt that the phenomenon of being ‘with woman’ is unfamiliar in the context of biomedical approaches to maternity care and perceived as of less value.

… *it’s [with woman] not supported in the majority of places where midwives work or models of care that midwives work in it’s not an acknowledged skill really. It’s not considered important, it’s not evidence based, it’s not scientific … it’s not supported and it’s not promoted as valuable* (P2).

Another midwife expressed frustration with communicating the importance of being ‘with woman’.

… *it’s a matter of, for us to be able to put it [with woman] in a language that doesn’t seem so tree hugging … what we do is both an art and a science, so how do you find that, for something that we know* (P3).

Midwives recounted a blurring of the lines of accountability when unknown practitioners enter the woman’s birth space and interrupt being ‘with woman’.

*We’re in labour ward but we have other members of staff on labour ward. So you have the coordinator and you have the GPs [general practitioners] and you have the specialist obstetricians and sometimes I think when that model* [medically-led care] *comes into my room with my woman that affects me being with woman* (P4).

This contrasts with descriptions of when labour and birth occurs at a birth centre, or the woman’s home: *Your role is going to be different when you’re at home and everything’s going well, she’s in the zone and things are really smooth then you’re really her support, facilitator and guardian, making sure it all goes according to plan* (P9)*.*

Midwives also reflected on the limitations of service availability such as when there were more women wanting maternity care in a ‘known midwife’ model than there were midwives to provide it.

*I think that one of the other things that’s really impacted upon my ability to be able to be there with women is that the demand for our services is outstripping the supply and it’s very hard to say to a woman I’m sorry but you haven’t made it on the programme … that’s been a challenge not to be able to give every woman that service* (P3).

Another limitation was seen as the rationalising of antenatal visits in some services.

It’s continuity of care of course but then we see them at 15 weeks and then we don’t see her, then ‘til 24 weeks. So it could be better, could be earlier … for some women they need more time to build the relationship (P7)*.*

#### Midwives as the ‘vehicle’ for ‘with woman’

Midwives described the potential for personality clashes to impact on their ability to be ‘with woman’: *Personality … would be the only thing that would come up, but in my experience, in the antenatal period this has been resolved* (P2). Midwives recognised that their own personality may, in some circumstances provide an obstacle: *I think that your own personality causes a barrier to be honest … some women you don’t particularly gel with* (P10). Midwives offered insights into how they personally can impact on being ‘with woman’ and shared openly how this happens and strategies to overcome these challenges. One insight related to personal pressures affecting mood.

*When you’re not feeling up to it and you have to put yourself in the right mood but as soon as it [labour] starts it gathers momentum and becomes easier and flows really nicely … if you’re not in the right space then it doesn’t take much to flick you over and see the power and the beauty of what’s before you and help to make that happen [being with woman] if you’re in your ‘with woman’ zone it helps the woman to be in her zone* (P9)*.*

Midwives spoke about how their own fears from past personal or professional experiences, influenced their ability to be with woman and how they learned to overcome this: … *we project our own fears and our own baggage into what the woman is saying but this is not your journey this is her journey … I learned a lot about, not projecting what I felt or what experience* (P7). Fear of ridicule from colleagues was also acknowledged as a challenge: *I think then there’s the scared of being told that they’re weird or there’s no science behind that, like being fobbed off because they do they make you feel unintelligent or because you believe in that [being with woman]* (P2).

#### Women who are not known or don’t want to be ‘known’

Despite continuity of care being a key characteristic of the ‘known midwife’ model, a midwife may be called to care for a labouring woman in another team or to care for a woman booked to a privately practising midwife who is not available. Midwives reflected on the impact of being ‘with woman’ during labour and birth for a woman they have not provided antenatal care for.

… *it is different but … even if we haven’t met the woman specifically, we all know something about each other’s women … we’re all continuously talking so most of the time even if I haven’t met the woman I know something of her and something of her wishes* (P5).

Although, not commonly reported in this research, midwives described how a woman can dismiss and not recognise the relationship opportunity in a ‘known midwife’ model. Women who may decline ‘relationship’ are still offered respectful care during labour and birth: … *there’s other women who as long as you’re clinically there and doing everything right and everything’s getting addressed they don’t have that ‘with woman’ connection … doesn’t matter which midwife they had* (P4). Another midwife shared a scenario where: … she just was a woman who actually she didn’t want me in her space, she didn’t want me to be involved, she just wanted me to do the birth … she didn’t want her husband or … no input from him and you know she said just don’t f***ing touch me (P6). Midwives acknowledged how being ‘with woman’ in these situations means being respectful of a woman’s wishes by providing care less focussed on the relational aspects of care.

## Discussion

The findings from this study confirm the importance of respectful, professional relationship to the practice of being ‘with woman’ which enhances midwives’ ability to provide woman – centred care. Although being ‘with woman’ is a phenomenon with a philosophical origin, the application of this practice impacts midwives, the profession of midwifery as well as women and their families. The challenges described by midwives also offered insight into how being ‘with woman’ intersects with various models of care and places of birth.

### Relationship is key

Western Australian midwives’ experiences of being ‘with woman’ during labour and birth in the context of the ‘known midwife’ model is permeated by the trusting relationship between the midwife and woman that is central to this model of maternity care. Midwives’ ability to be ‘with woman’ during labour and birth is enhanced by the knowing and trust that comes out of the relationship developed in the antenatal period [45]. The various embodiments of ‘knowing’ in midwifery practices have been written about in midwifery literature globally [46–48], the importance of the ‘knowing’ that comes from relationship is confirmed in our findings as highlighted visually in the word cloud generated from the interview transcripts (Fig. 2).

Because the relationship is developed in the antenatal period, being ‘with woman’ during labour and birth commences during pregnancy when relationships are being built. Midwives’ descriptions reveal new evidence that being ‘with woman’ in the ‘known midwife’ model is inextricably linked to whichever stage of the childbearing continuum the woman finds herself in. Being ‘with woman’ in the ‘known midwife’ model is not isolated to the labour and birth experience which is an important and new finding in this study. Although there are a small number of publications that refer to examples of being ‘with woman’ in the antenatal [21] or postnatal period [49], many of the writings that address midwives being ‘with woman’ predominantly refer to labour and birth [10, 50–52]. Further research is warranted into how being ‘with woman’ is developed during pregnancy and sustained across the childbirth continuum.

Historically, the concept of being ‘with woman’ has been criticised for being exclusive to others including the woman’s partner or family [53]. Our research findings confirm the theoretical writings from midwifery leaders that propose inclusion of the woman’s partner and family as an essential component of ‘with woman’ philosophy and practices offers important new evidence [18, 26, 54, 55]. Building knowledge around the phenomenon of being ‘with woman’ from the perspective of the partner and significant family members should also be a focus of future research.

### Woman centred care

Being ‘with woman’ during labour and birth in the context of the relationship that is formed in the ‘known midwife’ models, facilitates intuitive and individualised care placing the woman and her family at the centre of the care experience. Midwives’ descriptions of this align with what is understood in the theoretical domains about woman-centred care as being an integral element of the ‘with woman’ philosophy [1, 26, 56, 57] but is presented empirically for the first time here. Being ‘with woman’ in the ‘known midwife’ model transcends maternal risk and has been shown to act as a buffer to women whose clinical condition changes throughout pregnancy as well as labour and birth [58–60] . The relationship that develops in the ‘known midwife’ model enhances midwives’ ability to be ‘with woman’ by providing culturally safe care, particularly for Indigenous women or those from a culturally and linguistically diverse background. This is consistent with findings from other Australian authors that assert that the relationship in ‘known midwife’ models positively influences cultural responsiveness [61–63] .

### Impact of being ‘with woman’ in the context of the ‘known midwife’ model

Being ‘with woman’ during labour and birth in the ‘known midwife’ model impacts on midwives, the profession of midwifery, women as well as their partners and family. Midwives described their personal investment in professional practice as they gave from themselves in order to be ‘with woman’. This phenomenon of how the broader work of midwifery intersects with midwives’ emotions and humanity has been previously explored in works by Fenwick et al. [64] and Hunter and Deery [65]. The important and new finding of how being ‘with woman’ enhanced the confidence of the midwives is supported by Kennedy et al. [19] who describe the similar practice of midwives being ‘present’ during labour and birth as an essential feature of midwifery. The novel discoveries in this research confirm that the relationship that is formed in the ‘known midwife’ model provides an environment that enhances midwives’ ability to be reflexive in practice. In addition to this, the space that was created through the trusting relationship encouraged midwives to try new ways of being ‘with woman’ which supports professional development and future care of women, their partners and family. Although not specifically seeking to understand the phenomenon of being ‘with woman’, recent evidence suggests continuity models enable and motivate midwives to invest in and extend their practices contributing to greater satisfaction in women [58, 66].

The findings from this study emphasise the importance of being ‘with woman’ to the profession of midwifery and confirm that being ‘with woman’ contributes to identifying what it means to be a midwife. This debate has been raised in earlier writings as well as the findings of recent research [11, 66–68]. Importantly, midwives confirmed that being ‘with woman’ during labour and birth in the ‘known midwife’ model was rewarding and sustaining. Indeed, findings from recent research showed that midwives experienced greater satisfaction when working in models that support their professional philosophy [54, 69, 70]. Given the debate surrounding ‘with woman’ as being essential to being a midwife, research into midwives’ perceptions is recommended around whether midwifery care can be delivered in the absence of being ‘with woman’ within the context of different models of maternity care.

Midwives’ descriptions of the benefits, reported by women who received ‘with woman’ midwifery care during labour and birth in the ‘known midwife’ model included characteristics recognised in international literature such as guidance, respect, dignity and empowerment [19, 71, 72]. Midwives also highlighted that women who had previously experienced labour and birth care by an unknown midwife commented on the difference between the two models and appreciated the relationship with the midwife in the ‘known midwife’ model. Whilst maternal outcomes and satisfaction rates are consistently rated positively by women in continuity of care models [23], there is no current evidence that captures women’s experiences across different models of maternity care, which also warrants further research. Similarly, further research is warranted to explore midwives’ experiences of working in different models.

### Working through the challenges

Several factors challenged midwives’ ability to be ‘with woman’ during the intrapartum period within the ‘known midwife’ model context. A ‘systems’ approach to childbearing that favours standardisation over individualisation of care and ‘throughput’ over relationship is acknowledged as a known barrier to woman-centred care [73] in this study, it is also shown to impact the phenomenon of being ‘with woman’. Where ‘known midwife’ models of care are required to intersect with these systems either through formalised structures or places of birth this challenge becomes apparent [50]. This issue is not unique to the Western Australian maternity landscape and is supported in the writings of midwives from the USA, [74], UK, [75, 76] and Norway, [69].

Midwives acknowledge the human aspect of working ‘with woman’ and their families which highlights how personality or feelings of the midwife and woman may offer a challenge to being ‘with woman’. Midwives demonstrated their resilience by offering strategies to address the issue of not feeling ‘up to the task’ which offers the first, ever insight into how midwives reconcile the challenges and rewards of being ‘with woman’ The concept of ‘emotion work’ in midwifery is an emerging topic and focuses principally on the emotional impacts on midwives [64, 65, 77]. Further research is warranted on the impact of interpersonal constructs of personality clashes in maternity care. The realisation that some women do not want to be ‘known’ and prefer clinical and practical care over an experience founded in relationship during labour and birth has not previously been reported and should be explored.

### Strengths and limitations

A strength in this research study includes the collection of data from midwives currently working in a ‘known midwife’ model but who also had experience working across a range of ‘known midwife’ models and models where the midwife is not known to the woman. This focus enabled these WA midwives to reflect upon the intersection between being ‘with woman’ in the context of the ‘known midwife’ models including MGP and private practice. It could be asserted that self-selection into this study suggests that midwives recruited from ‘known midwife’ models may hold distinctive views of being ‘with woman’ compared to views of midwives working in other models of care and findings must be considered within this context. The richness of the data presented allows the reader to determine any potential transferability of the findings to other models of care.

## Conclusion

Our findings provide insight into midwives’ experience of being ‘with woman’ while providing care during labour and birth within the context of a ‘known midwife’ model. Being ‘with woman’ in this model is underpinned by a trusting relationship that influences not only the midwife and woman, but her partner, family and the profession of midwifery. Findings confirm some previously understood concepts about the phenomenon of being ‘with woman’ but also highlight new insight that has never been acknowledged from the previously overlooked but unique voice of midwives working in this model of care. Understanding midwives’ experiences of being ‘with woman’ as well as the challenges they face, offers the opportunity for service providers to explore innovative ways of facilitating ‘with woman’ care in this model. Insight into the applied practices of being ‘with woman’ in a ‘known midwife’ model adds valuable knowledge around a phenomenon that is central to the profession’s philosophy and practice.

## Abbreviations

ACM: Australian College of Midwives
CMP: Community Midwifery Program
KM: Known Midwife
MGP: Midwifery Group Practice
UK: United Kingdom
US: United States of America
WA: Western Australia
WW: With woman
